# Parental Factors Associated with Child Post-traumatic Stress Following Injury: A Consideration of Intervention Targets

**DOI:** 10.3389/fpsyg.2017.01412

**Published:** 2017-08-22

**Authors:** Anna E. Wise, Douglas L. Delahanty

**Affiliations:** ^1^Department of Psychological Sciences, Kent State University, Kent OH, United States; ^2^Northeast Ohio Medical University, Rootstown OH, United States

**Keywords:** child injury, parent, post-traumatic stress, intervention

## Abstract

Post-traumatic stress disorder (PTSD) symptoms are relatively common following pediatric traumatic injury and are related to poor long-term child outcomes. However, due to concerns regarding the efficacy of early child preventive interventions, and difficulty intervening with injured and medicated children soon after the event, it is not feasible to provide early psychological interventions to children exposed to traumatic injury. Parental PTSD symptoms and reactions to the child’s traumatic injury impact child outcomes and provide potential targets for early intervention to reduce child symptom development without involving the child. The authors conducted a review of the literature using Psycinfo and Pubmed research databases (publication years = 1990–2017) and identified 65 published studies relevant to the topic of the review. The present review considers parent factors [parenting styles, parental post-traumatic pathology (PTS), adaptive and maladaptive coping strategies, and communication regarding the traumatic injury] and their impact on child PTS. We focus specifically on factors amenable to intervention. We further review moderators of these relationships (e.g., child age and gender, parent gender) and conclude that it is unlikely that a one-size-fits-all approach to treatment will be successful. Rather, it is necessary to consider the age and gender of parent child dyads in designing and providing targeted interventions to families following the traumatic injury of a child.

## Introduction

Prior investigations of pediatric traumatic injury patients have reported relatively high rates of post-traumatic stress and comorbid behavioral and emotional disorders (Daviss et al., 2000; Winston et al., 2002; Landolt et al., 2003). Childhood stress-related pathology is associated with significant social impairments, cognitive deficits, poor academic performance, and increased risk for emotion-related disorders over the lifespan, underscoring the importance of early identification and appropriate intervention to reduce post-traumatic stress pathology (PTS: Reinherz et al., 1993; Berman et al., 1996; Famularo et al., 1996; Fletcher, 1996; Stallard et al., 1998; Lipschitz et al., 1999; Heim and Nemeroff, 2001). For the present manuscript PTS will refer broadly to post-traumatic psychological pathology including depression, anxiety, PTSD, etc., while PTSS will refer specifically to PTSD symptoms. Although a number of pre-, peri-, and post-trauma factors have been associated with increased risk for the development of PTSS in child trauma survivors, together these risk factors account for a relatively small percentage of the variance in subsequent PTSS symptoms (Trickey et al., 2012). One factor that must be taken into account in child trauma studies is the influence of family factors and parental responses to a child’s trauma on a child’s recovery (Bowlby, 1969; Rolland, 1994). Parents or guardians of children exposed to traumatic injury are equally likely as the child, if not more so, to develop PTS in response to their child’s injury, and parent reactions can impact the child’s symptoms and recovery (Landolt et al., 2003; Nugent et al., 2007; Cox et al., 2008). The present paper will review literature examining parental factors (parental PTS, parental coping, and household communication regarding the event) and their influence on the development of PTS– in child injury survivors. Specifically, this paper focuses on factors amenable to early intervention in order to provide a comprehensive understanding of potential targets for interventions with parents. Given challenges with intervening with seriously injured, and oftentimes heavily medicated, children soon after a traumatic injury, we also focus on factors amenable to intervention in parents that do not require active child participation. Thus, parent–child conflict and other relationship-related risk factors are not included in the present review. Finally, research had demonstrated that the impact of parental and familial factors on child symptom development is complex, often differing depending upon child gender, parent gender, and child age/development (Morris et al., 2012; Gutermann et al., 2016). Thus, we will also consider parent and child demographic variables (age and gender) that may interact with the abovementioned factors in order to impact child recovery.

## Methods

The authors conducted a review of the literature using Psycinfo and Pubmed research databases. The following search terms were used: “child traumatic injury,” “pediatric injury,” “post-traumatic stress symptoms,” AND “parent influence,” “parent behavior,” “parent symptoms.” Following a review of relevant abstracts, the authors identified 65 published studies relevant to the topic of the review that were published between 1990 and 2017. Studies that defined child injury as an accidental, physical, acute injury was included. Children in the identified studies experienced motor vehicle accidents, neighborhood violence, accidental injury, and accidental injury requiring hospitalization. The authors also review some literature from other trauma populations (e.g., war, terrorist attacks, natural disaster, and pediatric cancer) in order to provide examples or theoretical rationale. The reviewed literature included samples of children ages 4–18 years old: however, most samples consisted of adolescent children. Child and family responses to the trauma were measured at varying time periods following the injury; in some cases immediately following the injury and at other times a few months to a year following the injury. We specify when the literature is referring to responses that are immediate or delayed.

## Post-Traumatic Stress Pathology (PTS) in Children Exposed to Traumatic Injury

Worldwide, injury is a leading cause of death, morbidity, and acquired disability among children (Peden, 2008). Twenty million children in the United States each year experience unintentional injuries, resulting in 8.7 million emergency room visits; 241,000 children annually are injured seriously enough to be hospitalized (Safe Kids United States, 2008). Mental health sequelae of traumatic injury range from transient distress to PTSD. According to the Diagnostic and Statistical Manual for Mental Disorders (DSM-5: American Psychiatric Association, 2013), PTSD includes symptoms of re-experiencing (i.e., intrusive thoughts, nightmares, flashbacks), avoidance of trauma-related stimuli (i.e., avoidance of trauma- related thoughts, feelings, or reminders), negative thoughts or feelings (i.e., negative thoughts or assumptions about oneself or the world, negative affect, feeling isolated), and trauma-related arousal and reactivity (i.e., irritability or aggression, hypervigilance, heightened startle reaction). A population-based cohort study found that injured youth aged 10–19 were at significantly increased odds of developing PTSS; 6–32% of injured children develop PTSD, and up to 20% develop sub-syndromal PTSD (Mirza et al., 1998; Stallard et al., 1998; Keppel-Benson et al., 2002; Kassam-Adams and Winston, 2004; Stallard et al., 2004; Landolt et al., 2005). As number of PTSS increase, other negative outcomes such as functional impairment, number of comorbid disorders, rates of comorbid major depressive disorder, and current suicidal ideation also increase linearly, underscoring the importance of understanding predictors of PTS even in acute injury samples with relatively low rates of full PTSD (Stein et al., 1997; Aaron et al., 1999; Schutzwohl and Maercker, 1999; Marshall et al., 2001).

## The Consequences of Childhood PTS

Childhood PTSD is associated with significant social impairments (Berman et al., 1996; Stallard et al., 1998); cognitive deficits (Bremner et al., 1995); poor academic performance (Reinherz et al., 1993); and a variety of comorbid disorders (Fletcher, 1996). Comorbid anxiety, depression, and alcohol and drug dependence are common in pediatric patients with PTSD (Famularo et al., 1996; Lipschitz et al., 1999; Giaconia et al., 2000). Moreover, chronic PTSD in children has been associated with persistent hormonal and neuroanatomical abnormalities and has been identified as a risk factor for the development of psychopathology in individuals who experience a subsequent trauma in adulthood (e.g., Lemieux and Coe, 1995; De Bellis et al., 1999a,b; Carrion et al., 2002). Given the chronic, debilitating course of pediatric PTSD (Lonigan et al., 2003), early intervention is necessary to address emergent psychiatric symptoms before they develop into a chronic syndrome. However, early interventions in seriously injured children present challenges, as injuries, medication, physical therapy and recovery can all impact ability and motivation to engage in early therapeutic approaches. Parental reactions to a child’s traumatic injury have been found to increase a child’s risk for PTS (reviewed below). Thus, the present paper focuses on early interventions that could be implemented with parents to reduce or buffer the development of PTS in child injury survivors.

## Challenges with Conducting Early Interventions for Injured Children

A family history of psychopathology, prior trauma, peri-traumatic distress and dissociation, and post-traumatic social support have all been identified as consistent risk factors for the development of PTSD/PTSS (for a meta-analysis, see Ozer et al., 2003). Research in pediatric trauma survivors has found that pre-existing depression and anxiety, perceived trauma severity, peri-traumatic distress, age, gender, socioeconomic status, and prior trauma experiences have all been related to child PTSS (for a meta-analysis see, Cox et al., 2008). However, these risk factors, independently and together, account for a relatively small percentage of the variance in subsequent PTS, and existing screeners are relatively poor at identifying individuals who would benefit from intervention (Winston et al., 2003; Kenardy et al., 2006; O’Donnell et al., 2008). Consequently, early identification of children at-risk for developing PTS following an injury has proven difficult. Further, given that only a minority of children exposed to traumatic injury develop PTS, intervening with all injury survivors is neither cost- nor labor-effective, as the majority of individuals who would receive an intervention would not have developed persistent symptoms (McNally et al., 2003). Early intervention approaches have also met with limited success, with research finding that early interventions with children are often ineffective and, in some cases, detrimental (McNally et al., 2003). Furthermore, as mentioned above, it can be logistically difficult to intervene with a child immediately following a traumatic injury. Therefore, addressing parental/familial risk factors soon after a child’s injury may be a more efficient and efficacious way to decrease child PTS.

## Family Functioning in Response to A Child’s Trauma

As opposed to adults who experience traumatic injury, children are not autonomous in their decisions to approach or avoid reminders of traumatic events. For instance, whether a parent continues to drive through an intersection where a child’s injury occurred is the decision of the parent, not the child. Thus, child trauma survivors are impacted by parental behaviors. The influence of parent and family factors must be incorporated into any study of children exposed to traumatic injury. Parental and familial/household responses to a child’s trauma impact the child’s recovery, and have been found to moderate early child risk factors.

### The Impact of Parenting Styles on Child PTS

A wealth of data have suggested beneficial effects of sensitive parental caregiving and adverse effects of negative parenting on child anxiety and depression symptoms (Krohne and Hocke, 1991; McCauley et al., 2001). Negative parenting styles of rejection, hostility and conflict have been associated with increased depression in children and adolescents (Garber and Flynn, 2001; McCauley et al., 2001; Mezulis et al., 2006), although poor maternal parenting may differentially impact boys vs. girls (McFadyen-Ketchum et al., 1996). It has been hypothesized that parents model responses and provide feedback concerning the causes, consequences, and meaning of negative events (Mezulis et al., 2006). In addition to negative parenting techniques evoking both direct and indirect influences on a child in general, parental responses to a child’s trauma can also impact the child’s recovery following a traumatic event. Research has specifically investigated how parenting styles or practices may influence the development of psychopathology in children following a traumatic event and has shown that the presence of a responsive, predictable, and nurturing caregiver is essential to a child’s healthy neurobiological recovery following a traumatic experience (Perry and Pollard, 1998). Cobham and McDermott (2014) found that altering parenting practices following a natural disaster, such as becoming more protective, less granting of autonomy, and communicating a sense of current danger following a disaster were associated with worse child outcomes. Alterations in parenting styles often prevent child re-establishment of normal life routine and activities following a traumatic injury (Williamson et al., 2017). A higher level of parental overprotection has also been identified as a risk factor for the development of PTS following a child traumatic event (Henry et al., 2004; Bokszczanin, 2008) perhaps because parental overprotection hinders child exposure to trauma-related material or activities which may maintain symptoms and prevent natural recovery (Williamson et al., 2017).

A recent meta-analysis of 14 studies with 4010 participants examined the impact of positive and negative parenting on child PTSS in child trauma survivors aged 9–16 (Williamson et al., 2017). Results revealed that negative parenting behaviors (e.g., hostility and overprotection) following child injury accounted for 5.3% of the variance in child PTSS development, while positive parenting behaviors (e.g., warmth and support) accounted for 2% of the variance in child PTSS development (Williamson et al., 2017). Thus, positive and negative parenting have consistent but small impacts on child PTSS development.

### Parental and Familial Variables Impacting Child Symptom Development

As parenting style accounts for such a small percentage of the variance in child outcome, we focus specifically on theoretically and empirically informed early parental/family responses to a child’s trauma that are particularly relevant to a child’s recovery following a traumatic injury. Specifically, we review the impact of parental PTS, adaptive and maladaptive coping strategies, and communication regarding the traumatic injury on the child’s risk for PTS. These processes are reviewed specifically because they have been identified as potential mechanisms through which child symptoms develop, and existing interventions have demonstrated promise at reversing or improving these negative processes.

As mentioned, given that research has identified that the impact of these factors may differ by parent gender, child gender, and child age/developmental stage, we briefly consider the extent to which gender of parent–child dyads and age of the child may impact intervention approaches.

## The Impact of Parental PTS on Child Recovery from Traumatic Injury

Child trauma survivors are not just affected by their own responses to trauma; parental response to the child’s trauma has been shown to consistently have an impact on child adjustment. Higher levels of general distress after a traumatic event may result in a parent being less available to their child during the early post-traumatic period (Schwartz et al., 1994). However, parental PTSS stemming from the child’s trauma have been more strongly related to the development of PTSD in children than general parental distress (Pelcovitz et al., 1998; Nugent et al., 2006), suggesting specific risk afforded by parental PTSS and underscoring the importance of examining familial influences and responses to the child’s trauma as moderators of adjustment in children exposed to trauma.

However, research examining the association between parent and child symptoms following a child’s trauma has produced equivocal results. Many studies have found a significant positive relationship between parental and child PTSS (de Vries et al., 1999; Daviss et al., 2000; Keppel-Benson et al., 2002; Landolt et al., 2005, 2012; Schreier et al., 2005; Nugent et al., 2006; Ostrowski et al., 2007; De Young et al., 2014; Halevi et al., 2016). Other studies; however, have found no relationship between parental and child PTSS (McDermott and Cvitanovich, 2000; Koplewicz et al., 2002; Landolt et al., 2003). One potential explanation for the equivocal nature of these findings concerns the methods of assessing PTSS. Studies in which parents’ report on both their and their child’s symptoms tend to find higher concordance of symptoms than studies in which the child reports on their own symptoms (de Vries et al., 1999).

An additional explanation for contrasting findings could be the timing of post-trauma assessments (Schreier et al., 2005). Studies examining acute distress levels soon after the trauma typically find no association between parent and child PTSS (Winston et al., 2002; Bryant et al., 2004), while studies looking at more persistent distress tend to find significant correlations (Koplewicz et al., 2002; Schreier et al., 2005). Studies including multiple assessment points suggest that initial symptoms in the child can impact subsequent symptoms in the parent (Koplewicz et al., 2002), and that acute symptoms of distress in the parent predict subsequent PTSD in the child (Daviss et al., 2000). Further, concordance of symptom reporting may develop over time, as Koplewicz et al. (2002) reported that the correlation between parental and child PTSS increased during subsequent follow-up assessments.

Parental PTSS has also been found to interact with child biopsychological risk factors post-trauma to increase risk for child PTSS. Parental PTSS moderated the relationship between early child biological risk factors and child PTSS 6-months following a child’s traumatic injury (Nugent et al., 2007). More specifically, parental (primarily maternal) PTSS moderated the relationship between in-hospital child cortisol levels and subsequent child PTSS, and between in-hospital child heart rate and subsequent child PTSS, such that higher parental PTSS interacted with child symptoms to predict higher child PTSS. These results suggest that children who were not at risk for PTSS based on initial biological responses to the trauma may develop PTSS due to their parent’s response.

Further, maternal PTS has been found to predict 6-months child PTSS following Cognitive Behavioral Therapy (CBT) treatment. Despite evidencing initial improvement during CBT, children whose mothers had higher depression levels subsequently had increased PTS compared to children whose mothers had lower levels of depression (Weems and Scheeringa, 2013). One possible explanation for the impact of parental PTS on a child’s recovery from a traumatic injury involves the extent to which parental PTS symptoms result in increased parenting stress. Parenting stress, but not parental emotional availability, has been found to have a significant indirect effect on the relationship between parental PTSS and child outcomes (Samuelson et al., 2016). Thus, parental PTS may decrease parental resources, resulting in increased parenting stress and contributing to the development of child PTS.

Given the consistently observed impact of parental PTS on child’s recovery following a traumatic injury, it is likely that simply treating a child’s PTS without addressing parent PTS would limit the efficacy of child intervention approaches. There are a number of efficacious interventions to address PTS in adults exposed to traumatic events [Prolonged Exposure Therapy (PE); Cognitive Processing Therapy (CPT); Cognitive Therapy (CT): Foa et al., 2008]. Although these therapies have been found to be effective in reducing PTS in a variety of trauma samples, research has not examined their efficacy in treating adults whose index trauma is the traumatic injury of their child. Also, research investigating the efficacy of early interventions [i.e., Critical Incidence Stress Debriefing (CISD); Psychological Debriefing (PD); Early Cognitive- Behavioral Interventions (E-CBT)] for traumatized adult samples has generally found that early interventions with components of E-CBT are more efficacious than CISD and PD interventions (Foa et al., 2008). Although, again, it is unclear whether these treatment approaches would buffer or prevent symptom development in parents of injured children. Additional research is necessary to determine whether established PTS treatments can mitigate symptoms in parents of injured children, as it is likely that addressing PTS in parents will have a positive impact on child PTS.

## Additional Parental Responses and Their Influence on Child Recovery

In addition to the impact of parental PTS on child recovery, research has revealed a number of other parental reactions to a child’s trauma that could buffer or increase risk for the development of PTS in child traumatic injury survivors. Specifically, the manner in which a parent copes with the child’s injury and aids the child in coping with the trauma may significantly impact child recovery.

### Protective Parenting Strategies- Promoting Adaptive Coping Strategies

Following a child’s injury, parents use many different types of coping strategies in order to encourage a healthy stress response in the child (Marsac et al., 2013). The development of child coping strategies is socialized through parental coaching and modeling of coping strategies (Miller et al., 1994); thus, the manner in which a parent copes with a child’s injury can directly impact the way that the child copes. Children are more likely to use emotional regulation coping strategies if a parent provided assistance with emotional processing of the traumatic event (Marsac et al., 2014). Therefore, parental coaching and modeling of adaptive coping strategies appears to be an effective means of promoting healthy child coping following a traumatic injury.

Parents can also improve child outcomes by providing and assisting with social support coping. Social support provided by family members has been found to be particularly protective against the development of PTSS following a child’s traumatic event (Stallard et al., 2001; Meiser-Stedman et al., 2006; Dixon, 2016, Unpublished). Children who reported more social withdrawal following traumatic injury reported higher PTSS symptoms; however, parents that helped their children cope following the trauma had children that were more likely to seek social support (Marsac et al., 2013). This suggests that providing adaptive coping modeling or coping assistance increases social support seeking behaviors which in turn protect against the development of PTSS.

### Disadvantageous Parent Coping- Maladaptive Coping Strategies

Whereas parental modeling of adaptive coping strategies buffers against the development of child PTS following trauma, maladaptive coping strategies modeled by parents are also learned and implemented by the child, resulting in persistent PTS. For instance, distraction coping, a form of avoidant emotional coping, has been associated with higher levels of PTSD in children/adolescents following a variety of traumas [i.e., unexpected death of a loved one, (Schnider et al., 2007); natural disaster (Prinstein et al., 1996); road traffic accidents (Stallard and Smith, 2007)]. Children are more likely to use distraction as a coping strategy if their parent also used distraction (Marsac et al., 2014).

Additional poor coping strategies such as maladaptive cognitive styles and negative cognitive appraisals (i.e., beliefs that one’s life will be permanently changed, the world is dangerous, etc.) displayed by parents can be transmitted to children through learning and modeling (Schwartz et al., 1994). Following traumatic injury a child may model and express maladaptive threat appraisals and trauma cognitions in parents (Nugent et al., 2006). Other trauma samples have also found evidence for the impact of parental cognitions and appraisals on child symptoms. In one cross-sectional study, parental reports of disaster-related pathology (i.e., cognitions and behaviors that are altered due to the disaster experience) were associated with child PTSS at 3-months post natural disaster (Cobham and McDermott, 2014). Further, parental appraisals of alienation and permanent change were associated with later development of PTSS in children who experienced the death of a sibling (Morris et al., 2013). Negative post-traumatic cognitive appraisals in a child have been found to account for up to 60% of the variance in PTSS (Stallard and Smith, 2007), underscoring the importance of addressing these thoughts and behaviors in parents and children following a child’s traumatic injury.

In sum, the development of maladaptive coping and cognitive appraisals in the child may be reduced by intervening with maladaptive parental coping and cognitive appraisals. Researchers have highlighted the importance of addressing parental distressing or dysfunctional beliefs that developed following a child’s trauma in an attempt to increase parental modeling, and child learning, of adaptive coping behaviors and positive cognitive appraisals (Dalgleish et al., 2005). Given the importance of positive coping and cognitive appraisals following a child’s trauma, parenting interventions should encourage and teach healthy parental coping strategies in order to increase the chances of these healthy coping strategies being taught to, and learned by, the child. Furthermore, given the importance of social support in buffering against the development of child PTSS, parental interventions should provide parents with information, including language to use, to help discuss healthy coping with their child and provide examples of healthy coping strategies in the parent and child. Therapeutic interventions should provide psychoeducation on the importance of social support in child recovery, and discussion of ways in which parents can provide and improve child social support.

## Future Intervention Considerations

In addition to standard psychotherapeutic approaches to increase positive coping and social support and decrease maladaptive cognitions and appraisals, more recent research has suggested the potential utility of examining oxytocin as an adjunct to traditional psychotherapy. Oxytocin has been found to promote better psychological outcomes through its calming effect and modulation of fear-related memories (i.e., decreased amygdala activation and decreased amygdala and fear-reactive brain region connectivity) (Langeland and Olff, 2008; Yatzkar and Klein, 2010). Animal models have demonstrated that maternal care is associated with increased oxytocin receptor binding in the brains of offspring; however, this was only found in female offspring (Francis et al., 2002). In adult human samples, oxytocin administration has been associated with decreased physiological responses to traumatic imagery (Pitman et al., 1993), fewer acute PTSS, lower intensity of recurrent thoughts about the event, and decreased negative emotions (Yatzkar and Klein, 2010). Further, oxytocin administration has also been associated with improved mood and increased wish for social interactions compared to a placebo condition (Yatzkar and Klein, 2010). As children typically identify a parent as their main source of social support following a trauma (Dixon, 2016, Unpublished), parental social support is a potentially important factor contributing to child recovery following injury. Therefore, oxytocin as an adjunct therapy may increase parent–child social bonding and, as a result, decrease child PTS. No studies have investigated the effect of exogenous oxytocin administered to parents on child outcomes following a traumatic event or the association between parental and child oxytocin levels following a traumatic event. More research is needed to determine if parental oxytocin administration could be a potential target to improve child symptoms, but existent research is suggestive.

## Familial/Household Communication Regarding the Traumatic Event

In addition to direct parental influences on a child post-trauma, child PTS can also be influenced indirectly by the extent to which families discuss the traumatic event after it occurred. This has been less explored than other parental factors; however, recent research suggests that the manner and extent to which families discuss the trauma can also impact child PTS development. For instance, children in families in which caregivers did not discuss the traumatic event or did not express confidence in the child’s safety had higher PTSS than families in which the traumatic event was discussed (Carpenter et al., 2015). Thus, household discussion of the traumatic event is worth considering as a potential target for interventions. Interventions should promote caregiver expression of the child’s safety and caregiver disclosure of their feelings regarding the traumatic event.

## Potential Moderators of the Impact of Parental Responses on Child PTS

As previously mentioned, the manner in which parents and children interact following a child’s traumatic injury is complex, with myriad variables affecting risk or resilience of the child. Although the parenting and family environment factors discussed to this point are strong candidates for intervention, it is likely that the influence of these factors differ depending on the gender of the parent–child dyad considered (mother–son, mother–daughter, father–son, father–daughter) and age/developmental stage of the child. The impact that parenting factors have on an 8 years old are likely very different from the impact that the same factors would have on an 18 years old. We next consider these moderating factors to aid in informing the development of more flexible therapeutic interventions that can be tailored to an individual family.

### Parent Gender

Parent gender has consistently been found to have a moderating effect on the relationship between parent and child PTS. The vast majority of studies examining the impact of parental responses to a child’s trauma on the child’s PTS have examined maternal influences. A recent meta-analysis of 35 studies revealed that parent gender moderated the association between parent and child PTSS after a child’s exposure to a traumatic event such that maternal PTSS had a larger effect size than paternal PTSS (Morris et al., 2012). However, parent gender did not moderate the relationship between parent depression and child PTSS.

Relatively few studies have examined the impact of paternal PTSS on child PTSS development, and the majority of these studies have examined samples consisting of families in which a child has received a chronic disease diagnosis. These studies have typically found higher PTSD incidence in mothers vs. fathers (Landolt et al., 2003; Kazak et al., 2004). However, relatively little concordance has been found in self-reported PTSS of different family members. Landolt et al. (2003) found that maternal and paternal symptoms were significantly correlated (*r* = 0.50), but child symptoms were not correlated with either parent’s reports. Similarly, Kazak et al. (2004) examined PTSS in families of childhood cancer survivors and found very low concordance among child survivors, their mothers, and fathers (concordance rates ranged from 0 to 5%). In a study of families who experienced the death of a child, maternal PTSS was related to the surviving sibling’s PTSS, while paternal PTSS was not directly related to sibling PTSS (Morris et al., 2016). However, paternal PTSS was indirectly related to child PTSS through positive parenting, such that lower PTSS in the fathers was associated with more positive parenting and less child PTSS. It is unclear whether these results would generalize to families who experience the traumatic injury of a child.

In sum, research suggests possibly differing impact of maternal vs. paternal symptoms on child PTSS, but more research is needed to increase confidence in these findings. Research on paternal influences is particularly needed. Finding that maternal and paternal reactions to trauma may differentially impact children suggests that differing intervention approaches may be necessary for mother–child and father–child dyads. Further, family-focused interventions involving both male and female caregivers with the child may be complicated, necessitating an understanding of the differing influences of maternal vs. paternal PTSS influences on the child.

### Parent Romantic Attachment

Research has also found that the extent to which a parent has a secure attachment to a romantic partner impacts a child’s recovery following a trauma. Specifically, Quas et al. (1999) found that having parents with fearful-avoidant romantic attachments was associated with omission errors (i.e., leaving out or denying information) or avoidance of specific traumatic memory details during recall of a traumatic medical event (reviewed in Alexander et al., 2002). Further, having a parent with an insecure romantic attachment was associated with higher child distress following a stressful event. Adult reports of insecure-avoidant romantic attachments have been associated with low social support seeking behaviors and emotional avoidance following an interpersonal stressor (Fraley and Shaver, 1998). Similarly, insecure-ambivalent romantic adult attachments are associated with more distress and the use of emotion-focused rather than problem-focused coping strategies in an attempt to decrease or regulate arousal (Fraley and Shaver, 1999). Thus, in addition to direct effects on child recovery, poor parental romantic attachments may also indirectly impact the child through parental negative coping.

The reviewed literature suggests that it may be beneficial for interventions to work on improving parental romantic attachment as a means of improving the child’s symptom development. In particular, components of family systems therapy could be used to improve the emotional attachment of parental romantic relationships (Kerr, 1981). Further, both parents, if available, should be involved in therapeutic treatment aimed at reducing or preventing child symptom development. Improving parental romantic attachment may also support child adaptive coping through learning and modeling positive social support seeking.

### Child Developmental Stage

The developmental stage of the child at the time of injury may interact with parental and familial responses to the injury and differentially impact child outcomes. For instance, research on mother and child pain tolerance found that the association between mother’s pain tolerance and pain avoidance, and child pain responses was particularly high for prepubertal vs. pubertal children (Tsao et al., 2014). This finding suggests that prepubertal children may be more likely to model parental responses to painful experiences. Much of the child trauma intervention research to date has taken a one-size-fits-all approach, administering the same treatments to samples of children with broad age ranges. However, the treatment literature has demonstrated that treatment efficacy varies as an effect of child age. A recent meta-analysis investigated child age as a potential moderator between child PTSS treatment and child symptom outcomes (Gutermann et al., 2016). They found that child interventions targeting PTSS are more effective for older children and adolescents. One interpretation of this finding is that effective child treatments may include cognitive components that are difficult for younger children to grasp and implement (Gutermann et al., 2016). Thus, focusing on parental reactions to a child’s traumatic injury may be a particularly efficacious way of preventing or reducing PTS in younger children, for whom child-focused interventions have been less effective.

## Summary/Conclusion

The present review aimed to organize and synthesize the existent literature concerning possible targets for parent interventions that could be conducted soon after a child’s traumatic injury to reduce or prevent the development of child PTS. As mentioned, existing screeners aimed at identifying at-risk children following an injury are relatively poor and therefore, are not clearly identifying children in need of early intervention. Early interventions with children have had limited success, at times producing ineffective and even detrimental effects. Perhaps intervening with children who would have naturally recovered from the event may increase PTS symptoms in the child. For these reasons, and the fact that interventions with recently seriously injured children are logistically difficult, we propose that early parental interventions may be an efficacious way of impacting child PTS following a traumatic injury.

Effective interventions exist to promote positive parenting techniques [e.g., Triple P- Positive Parenting Program (Sanders, 1999); Behavioral Family Systems Therapy (BFST: Robin and Foster, 1989)]. However, given that parenting accounts for such a small percentage of the variance in child PTS, intervening with more proximal parental reactions to the child’s trauma that have been found to increase a child’s risk for persistent PTS may have a larger impact on promoting successful child recovery following a traumatic injury. Much research has demonstrated the importance of a healthy caregiver-child relationship for an adaptive stress-response following a traumatic event. The present review underscores the importance of treating parent PTS following a child’s traumatic injury. Parent PTS is common following the injury of a child, and parent PTSS has been found to relate to child PTSS and interact with other risk factors to increase PTS risk in a child. More specific targets of parental interventions were also reviewed to aid in prioritizing targets for parent interventions aimed at reducing child symptom development. Parental trauma-related threat cognitions and appraisals, specifically appraisals and cognitions related to alienation and permanent change, were found to particularly relate to child PTS development. Intervening with these appraisals and cognitions in the parents may prevent the modeling and learning of these negative appraisals and cognitions in children. This can be accomplished through psychoeducation in which parents are taught specific ways in which maladaptive trauma-related cognitions and appraisals may influence parenting practices and characteristics (i.e., becoming less protective, less granting of autonomy, and communicating a sense of current danger).

Our review also highlighted some important protective parent responses (e.g., parent coping assistance, emotional processing assistance, modeling and encouragement of social support seeking behaviors) that were associated with child psychological resilience following a stressful event. These characteristics and strategies should be taught and encouraged while maladaptive strategies such as parental distraction as a coping strategy, and other previously mentioned maladaptive trauma-related cognitions and appraisals should be decreased or discouraged.

The present review also underscored the importance of discussing, in an emotionally open, supportive, and caring manner, the traumatic event within the family. Avoiding discussing traumatic memories should be discouraged and skills training should involve emotional expression, engaging in activities related to the traumatic event, and encouraging disclosure of feelings regarding the traumatic event.

The present review has highlighted various independent targets for intervention for preliminary consideration as therapeutic components. Further research is needed to understand how these targets may be related to one another and how they may interact to contribute to the development of child PTS. Research is further needed to develop these individual targets into a cohesive intervention program. This research should also investigate the scheduling of targets to determine initial and secondary targets for intervention.

Finally, it is apparent that there are many moderators of the relationship between parent and child responses to the child’s traumatic injury. Research clearly indicates that the development of family focused interventions must take into account parent gender, child gender, and developmental stage of the child. Given the impact that these moderators have on child recovery post-injury, it is highly unlikely that a singular, one-size-fits-all approach to treatment interventions for parents of injured children will be effective at reducing or preventing child symptoms. Instead, future research powered to examine these 3-way interactions (parent gender × child gender × child age/developmental stage) is needed to inform intervention content for differing parent–child relationships.

There are several limitations to this review. First, given the wish to include only variables amenable to intervention, the review has a relatively narrow focus concerning factors associated with child PTS. Thus, we do not intend for this to be a cumulative review of the current literature on factors influencing child PTSS/PTSD development. Also, the review largely focused on pediatric traumatic injury samples, and results may not be generalizable to other ages or trauma samples. Finally, it is important to examine and consider trauma type (i.e., interpersonal vs. non-interpersonal) when selecting individualized intervention approaches, as interventions may be differentially efficacious for different types of child traumas. Examination of the appropriate match between trauma type and intervention approach was beyond the scope of the present review, but it is important that future research consider how differing trauma types may respond to differing therapeutic approaches. Despite these limitations, the present review provides a synthesis of the literature to inform research that aims to develop early interventions with parents of children who experienced a traumatic injury.

## Author Contributions

All authors listed have made a substantial, direct and intellectual contribution to the work, and approved it for publication.

## Conflict of Interest Statement

The authors declare that the research was conducted in the absence of any commercial or financial relationships that could be construed as a potential conflict of interest. The reviewer NV and handling Editor declared their shared affiliation, and the handling Editor states that the process nevertheless met the standards of a fair and objective review.
